# Comparison of Pregnancy Outcomes of History-Indicated and Ultrasound-Indicated Cervical Cerclage: A Retrospective Cohort Study

**DOI:** 10.1155/2023/8782854

**Published:** 2023-01-09

**Authors:** Joseph Ifeanyichukwu Ikechebelu, Cyril Chukwudi Dim, Boniface Chukwuneme Okpala, George Uchenna Eleje, Ngozi N. Joe-Ikechebelu, Divinefavour Echezona Malachy, Chinedum Mark Nnoruka, Louis Anayo Nwajiaku, Princeston Chukwuemeka Okam, Innocent Chigozie Albert, Augusta Nkiruka Okpala, Emeka Philip Igbodike

**Affiliations:** ^1^Department of Obstetrics and Gynaecology, Nnamdi Azikiwe University, Teaching Hospital, Nnewi, Anambra State, Nigeria; ^2^Department of Obstetrics and Gynaecology, Nnamdi Azikiwe University, Awka, Nigeria; ^3^Life Specialist Hospital, Nnewi & Life International Hospital, Awka, Anambra State, Nigeria; ^4^Institute of Maternal and Child Health, University of Nigeria, Enugu Campus, Nigeria; ^5^Department of Obstetrics and Gynaecology, University of Nigeria Teaching Hospital, Ituku-Ozalla, Enugu State, Nigeria; ^6^Department of Community Medicine & Primary Health Care, Faculty of Medicine, Chukwuemeka Odumegwu University, Awka, Anambra State, Nigeria; ^7^Social Dimensions of Health Program (INTD), School of Public Health and Social Development, University of Victoria, British Columbia, Canada; ^8^Department of Family Medicine, Nnamdi Azikiwe University Teaching Hospital, Nnewi, Anambra State, Nigeria; ^9^Department of Obstetrics and Gynaecology, Havana Specialist Hospital, Surulere, Lagos, Nigeria

## Abstract

**Background:**

Cervical cerclage is the procedure of choice for preventing preterm delivery due to cervical insufficiency. The indication for its application may be based on the woman's reproductive history, findings at ultrasound, or clinical findings on vaginal examination. Pregnancy outcomes from these indications are variable according to the available literature.

**Objective:**

To compare the effectiveness and reproductive outcomes (miscarriage, preterm birth rates, and birth weights) of McDonald's cervical cerclage after history-indicated and ultrasound-indicated cervical cerclage in pregnant women.

**Methods:**

The retrospective cohort study was conducted at Life International Hospital Awka, Nigeria and Life Specialist Hospital Nnewi, Nigeria. Pregnant women, who had a McDonald's cervical cerclage performed due to either history or ultrasound indication between January 1, 2011, and December 31, 2020, were included in the study. Women with multiple pregnancies and those with physical examination-indicated or emergency cerclages were excluded. The main outcome measures included the prevalence of cervical cerclage, miscarriage, and preterm delivery rates. Outcomes were compared between groups with the chi-square test, Fisher's exact test, or Student's *t* test. *p* value of < 0.5 was set as significant value.

**Results:**

Overall, during the study period, 5392 deliveries occurred in the study sites, of which 103 women had a history-indicated or ultrasound-indicated cervical cerclage. This resulted in a 1.91% prevalence rate for history-indicated and ultrasound-indicated cervical cerclage. Of these, 68 (66%) had history indicated, while 35 (34%) had ultrasound-indicated cerclage. There was no difference in the sociodemographic characteristics of both groups. Both groups had similar miscarriage rates: 1.18 in 1000 and 1.04 in 1000 deliveries, respectively (RR 1.160, 95% CI: 0.3824 to 3.5186, *p* = 0.793). There was more preterm delivery in history-indicated cerclage than ultrasound-indicated cervical cerclage (26.50% vs. 17.10%; *p* = 0.292), though the difference was not statistically significant. The ultrasound group had a higher average birthweight than the history group (2.67 ± 0.99 vs. 2.53 ± 0.74). However, this difference was not statistically significant.

**Conclusion:**

The effectiveness and reproductive outcomes (miscarriage, preterm birth rates, and birth weights) of pregnant women with cervical cerclage due to history-indicated and ultrasound-indicated cervical cerclage appear similar. When needed, cervical cerclage should be freely applied for cervical insufficiency, irrespective of the type of indication.

## 1. Introduction

Preterm birth (PTB) is one of the leading causes of neonatal and perinatal morbidity and mortality worldwide, and it accounts for a significant global health burden [1, 2]. It is more prevalent in low- and middle-income countries, with 15 million cases encountered globally every year. Sub-Saharan Africa accounts for approximately half a million neonatal deaths due to PTB [3, 4].

The aetiology of PTB is still of great research interest worldwide; however, it has been hypothesized that it is initiated by multiple mechanisms. Cervical insufficiency (CI), among other causes, is one of the major causes of preterm delivery. Some researchers define it as painless dilation of the cervix in the absence of uterine contractions, resulting in second or early third trimester delivery [5, 6] and passage of a size 8 Hegars dilator without resistance [6]. Its incidence is 1% of the world's obstetric population [7] and 8% in women with second trimester pregnancy loss [8]. Congenital abnormalities of the genital tract, connective tissue disorders, and trauma to the cervix are the most common causes of cervical insufficiency.

Cervical cerclage is a surgical procedure of choice for the treatment of CI that can be performed in an attempt to maintain the structural integrity of the cervix to prolong gestation and improve obstetrical outcomes [9]. It may be classified as history-indicated, ultrasound-indicated, and physical examination (clinical)-indicated cerclage [6, 10]. History-indicated cerclage is usually performed electively at early gestation (between 14 and 16 weeks) based on the previous history of midtrimester pregnancy loss. The Royal College of Obstetricians and Gynecologists (RCOG) recommends that history-indicated cerclage be offered to women with singleton pregnancies and three or more previous preterm births [11]. The effectiveness of history-indicated cervical cerclage has been studied with mixed results [12]. Ultrasound and clinical indicated are the major indications for an emergency cerclage, which can be placed up to 24 weeks of gestation and are indicated when there is a visibly dilated cervix on speculum examination or if there has been an unexpected finding of a shortened cervix or funnelling of the cervix on routine ultrasound examination [13].

The success of these cerclage procedures in preventing miscarriage or preterm delivery may be affected by a range of clinical parameters, patient characteristics, and indications for cerclage insertion. We conducted this study to test the hypothesis that more miscarriages and preterm deliveries occur following cervical cerclage due to history indicated rather than ultrasound-indicated cerclage. We compared the pregnancy outcomes (miscarriage rate, preterm delivery rates, and birthweight) of history- and ultrasound-indicated cervical cerclages.

## 2. Materials and Methods

### 2.1. Study Site

The study sites were Life International Hospital Awka (LIHA), Nigeria and Life Specialist Hospital, Nnewi (LSHN), Nigeria. Both LIHA and LSHN are private specialist hospitals that offer specialized care in obstetrics and gynecology. Both are referral centers for obstetrics cases from the subregion.

### 2.2. Study Design

It is a retrospective cohort study.

### 2.3. Study Population

Pregnant women who underwent cervical cerclage at the LIHA and LSHN between January 1, 2011 and December 31, 2020. Inclusion criteria: all the pregnant women with singleton pregnancies who underwent elective cervical cerclage using the McDonald technique within the study period were reviewed. Exclusion criteria: all the pregnant women who had medical disorders and multiple pregnancies were excluded. Those pregnant women who had a physical examination-indicated, emergency, or rescue cerclage were excluded.

### 2.4. Study Procedure

The main theatre, labor ward, and obstetrics theatre records of the hospitals were reviewed to identify patients who underwent a cervical cerclage procedure during the study period. The patients' case records were retrieved from the hospital record department after obtaining permission from the ethics committee. The patients' sociodemographic information, booking status, indication for the cervical cerclage, and gestational age at which the cervical cerclage was inserted were retrieved from the patients' case notes. The indications for cerclage were divided into two groups: group (1) history-indicated cervical cerclage and group (2) ultrasound-indicated cervical cerclage.

History-indicated cerclage is defined as a cervical cerclage placed usually at the end of the first-trimester based solely on a poor prior obstetrical history of two or more second-trimester pregnancy losses due to painless dilatation. Ultrasound-indicated cervical cerclage is defined as a cerclage placed in women on transvaginal ultrasound with an identifiable short cervical length (less than 25 mm) without cervical dilatation [14, 15].

The cervical cerclages were all inserted by 3 obstetricians in the hospital using McDonald's method under paracervical block with sedation or total intravenous anaesthesia (TIVA) with ketamine at the end of the first trimester for history-indicated cerclage and at the time of diagnosis for ultrasound-indicated cerclage. A 5 mm braided polyester fibre (Mersilene®) nonabsorbable fibre tape was used for all the patients. The study groups were compared in terms of clinical characteristics, pregnancy, and neonatal outcomes.

### 2.5. Sampling Technique

This was a nonrandom sampling approach. All available case files were reviewed.

### 2.6. Study Outcome Measures

Study outcome measures were cervical cerclage prevalence rate, miscarriage rate, preterm delivery rate, and neonatal birthweight.

### 2.7. Statistical Analyses

The data were entered into an Excel 2016 spreadsheet (Microsoft Corporation, Redmond, WA, USA) and subsequently imported into Statistical Package for the Social Sciences (SPSS) Version 28.0 (IBM Corp., Chicago, IL, USA). Continuous variables were compared using the student's *t*-test when normally distributed or the Mann–Whitney *U* test when they were not normally distributed. Categorical variables were compared between groups by the chi-square or Fisher's exact test. Values of *p* < 0.05 were considered to be statistically significant.

### 2.8. Ethical Approval

The study was approved by the Medical Research Ethics Committee of the Chukwuemeka Odumegwu Ojukwu University Teaching Hospital (COOUTH), Awka, Nigeria (date of approval: August 20, 2021; reference number: COOUTH/CMAC/ETH.C/VOL.2/FN:04/0045).

## 3. Results

**Figure 1:**
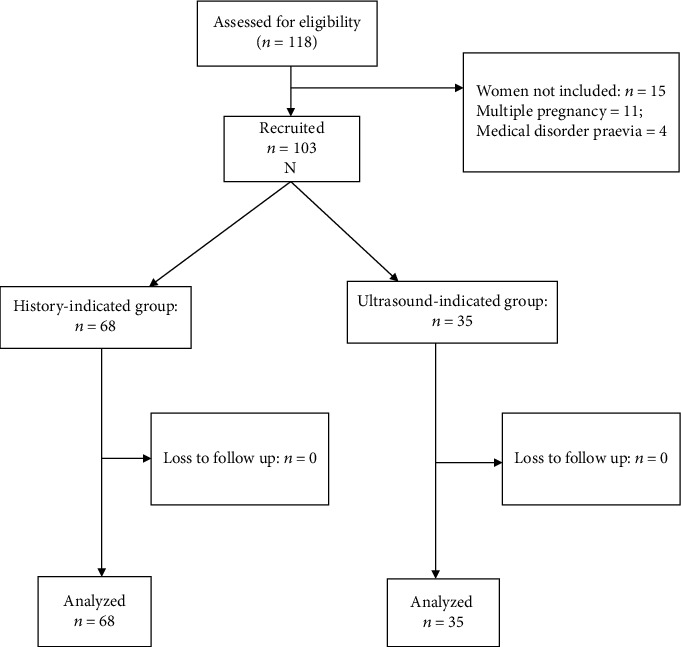
Study flow chart.

Overall, between January 1, 2011, and December 31, 2020, 5392 deliveries occurred in the study sites, of which 103 had cervical cerclage, exclusively for history-indicated and ultrasound-indicated cervical cerclage. This gave a prevalence rate of cervical cerclage of 1.91%, or 19.1/1000 births, for history-indicated and ultrasound-indicated cervical cerclage. The 103 participants were grouped into history indicated cervical cerclage 68 (66.0%) and 35 (34.0%) ultrasound indicated cervical cerclage. The flowchart is shown in Figure 1. The sociodemographic distribution of the participants showed that there was no significant difference between the two groups in terms of maternal age (*p* = 0.12), level of education (*p* = 0.061), marital status (*p* = 0.48), parity (*p* = 0.14), and body mass index (*p* = 0.77), as shown in Table 1. The gestational ages of cerclage insertion for history (14.43 ± 4.32 weeks) and ultrasound-indicated cerclage (14.71 ± 3.59 weeks) were similar (*p* = 0.57) as represented in Table 2. The Shapiro-Wilk tests showed that gestational age at insertion was not normally distributed (*p* < 0.05); hence, the Mann–Whitney *U* test was used for comparison, which showed that there was no significant difference in the mean gestational age at insertion across indication groups (*p* > 0.05).  Table 3 shows that the mean cervical lengths of both groups (history vs. ultrasound indicated) before cervical cerclage insertion are comparable (2.16 ± 0.29 vs. 2.20 ± 0.60, respectively).

Pregnancy outcomes are shown in Table 4. Both groups had similar miscarriage rates: 1.18 in 1000 and 1.04 in 1000 deliveries, respectively (RR 1.8571, 95% CI 0.5255 to 6.5629, *p* = 0.34). There was more preterm delivery in history indicated cerclage than ultrasound indicated (26.50% vs. 17.10%), though this was not statistically significant (*p* = 0.1181). There was also no statistically significant difference in term delivery (*p* = 0.59). The average birthweight was higher in the ultrasound-indicated group than in the history group. However, this difference was not statistically significant (*p* = 0.48), Table 5.

## 4. Discussion

Studies on interventions for preventing preterm births in women with a high risk of PTB are particularly relevant in low- and middle-income countries, where perinatal morbidity and mortality rates are high. This retrospective cohort-based hospital study has made an important contribution to existing knowledge about cervical cerclage outcomes based on indications of cervical cerclage placement. We compared pregnancy outcomes following two different indications for cervical cerclage: history indicated and ultrasound indicated. We found that there was no statistically significant difference in the pregnancy outcomes of both groups.

The cervical cerclage rate (for history-indicated and ultrasound-indicated cervical cerclage) in this study was 1.91% or 19.1/1000 births, which was comparable to other studies conducted in Croatia by Planinić-Radoš et al. [16]. They conducted a 16-year multicenter retrospective study on the incidence of cervical cerclage and preterm birth rates. In contrast to our study, they included those who had emergency cerclage in theirs. Although the number of patients undergoing emergency cervical cerclage is usually small, including it in their study may have influenced the incidence, while a population-based study by Lu et al. in Australia on the increasing incidence rate of cervical cerclage in pregnancy showed an increased rate of 3/1000 births to 3.8/1000 births over a 10-year period [17]. They did not state the inclusion or exclusion criteria for their study. This might have affected their outcome, while Osemwenkha et al.'s 6-year study of cervical cerclage in a Nigerian tertiary hospital revealed a lower incidence of 0.78% (7.8/1000 births) [18]. Only 3.3% of their study population had emergency cerclage, buttressing the point that emergency cerclage may have little effect on the incidence of cervical cerclage.

Our study showed that more participants had cervical cerclage due to history indication than ultrasound indication, though this was not statistically significant. This finding is consistent with previous research, which found that history-indicated cervical cerclage was more common than other indications [10, 17, 19]. This could be because the majority of participants who underwent cervical cerclage because of a previous history of midtrimester pregnancy will most likely present to the hospital for early pregnancy care in their subsequent pregnancies, increasing the incidence. Also, since the first-trimester ultrasound is now generally accepted as a routine investigation in obstetrics in most low- and middle-income countries, the incidence of ultrasound-indicated cerclage is expected to rise. However, an ultrasound examination is not readily affordable for all women on a routine basis due to its high cost in low-income countries. Nevertheless, we recommend routine first-trimester transvaginal ultrasound scans for all obstetrics patients in the study center; some pregnant women present late for booking, hence missing those who may be diagnosed by early ultrasound.

Pregnant women in the ultrasound-indicated cervical cerclage, surprisingly had a higher percentage of term delivery than those that had history-indicated cervical cerclage. This difference, though, was not statistically significant (*p* > 0.05). Many other studies, contrary to our findings, have found that women who had cervical cerclage for reasons other than history had more term births than those who had it for reasons other than ultrasound [17, 19]. Participants in our study had first-trimester transvaginal ultrasound; hence, the diagnosis of cervical insufficiency was made early, necessitating an early cervical cerclage intervention done in the early second- trimester. Application of cerclage at this stage might have increased the success rate of ultrasound-indicated cerclage. To buttress our point further, Golbasi et al. evaluated the effectiveness and perinatal outcomes of history-indicated, ultrasound-indicated, and physical examination-indicated cerclage. According to them, the gestational age at which the cervical cerclage was inserted in the history indicated cerclage was significantly lower than that of the ultrasound indicated cerclage [19], which contradicts our findings. They argued that the cerclage in the history indicated group may have been inserted prior to any cervical change, thereby prolonging the pregnancy [19]. We, therefore, believe that the time of insertion of the cervical cerclage may be a major contributor to the pregnancy outcome. The average gestational age of cerclage insertion due to transvaginal ultrasound diagnosis of cervical insufficiency in our study was 14.7 weeks. This is surprisingly earlier than what was reported in previous studies, where cerclage insertion due to ultrasound indication was at midtrimester [17, 19]. This is because we routinely recommend first trimester ultrasound for our patients, which resulted in the early application of cerclage in those whose cervixes were found to be short.

Interestingly, our study found no difference between the two groups in terms of miscarriage and preterm delivery (*p* = 0.793 and *p* = 0.292, respectively). This finding was consistent with the findings of Suhag et al. [20], who compared the incidence of preterm birth between the two groups. They found that after adjusting for confounders, the rate of spontaneous preterm birth at less than 37 weeks of gestation was similar between the transvaginal ultrasound cervical length screening and history-indicated cerclage groups (36.8% compared with 43.8%; adjusted OR 0.77, 95% CI 0.47-1.45 ^19^. In their study, Golbasi et al. [19], found that patients who underwent a physical examination indicated cerclage have a higher risk of PTB than the history-indicated and ultrasound-indicated cerclage. Similarly, they found no difference between history indicated and ultrasound indicated. This result might be due to the similar mean gestational age at cerclage insertion for both groups (14.43 ± 4.32 weeks vs. 14.71 ± 3.59 weeks) in our study. This buttresses the fact that early diagnosis and treatment of cervical insufficiency are the keys to preventing some of the perinatal complications due to PTB associated with cervical insufficiency.

The average birthweight in the history indicated was lower than the ultrasound-indicated group (2.53 ± 0.74 vs. 2.67 ± 0.99). However, this difference was not statistically significant. This result differs from that of Golbasi et al.^19^ (2.500 ± 0.967 vs. 2.645 ± 0.814). They also found in their study a lower birthweight (1.912 ± 1.232) for those that had emergency cerclage [19]. In their retrospective study of pregnancy outcomes and factors affecting the clinical effects of cervical cerclage when used for different indications, Chen et al. discovered similar birthweight between those who had cervical cerclage due to history indication and those who had emergency cerclage [14]. This is not surprising, as most of the patients who had emergency cerclage delivered at an earlier gestational age than those who had elective cerclage. This buttresses the point further that early diagnosis of cervical insufficiency and application of cervical cerclage improve pregnancy outcomes.

The main strength of the study was the detailed information about miscarriage and PTB characteristics, which was collected directly from hospital records. Cervical cerclage is a common procedure for obstetricians and gynecologists, so it had to be studied retrospectively using hospital records. All the patients had the same treatment protocols and a uniform surgical technique in the study center. Moreover, this retrospective study did not require consent from the participating women; thus, all identified cases were included (i.e., there was no selection bias). However, this provided an opportunity to explore every case thoroughly. The major limitation of this study is that it is retrospective in nature. Further, some of the associated factors for preterm labour and miscarriages were not controlled in this study. However, the two groups have similar demographic characteristics; therefore, the effects of these associated factors on the impact of this study may not be much.

## 5. Conclusion

In this study, we compared the effectiveness and perinatal outcome of cervical cerclage based on the indication. We found that there is no difference in the perinatal outcome of history-indicated and ultrasound-indicated cervical cerclage. We recommend that consideration be given to the early application of cervical cerclage before cervical changes that will lead to PTB have occurred. Every pregnant woman should undergo an early transvaginal ultrasound to identify those who will need cerclage insertion. Also, a large structured prospective randomized controlled trial should be conducted before generalizing the outcome of this study.

## Figures and Tables

**Table 1:** Distribution of sociodemographic variables among the indication groups.

		History *n* (%)	Ultrasound *n* (%)	Total *n* (%)	*X* ^2^	*p* value
Age range	24-34 years	36 (52.9)	20 (57.1)	56 (54.4)	4.18	0.12
	35-45 years	29 (42.6)	10 (28.6)	39 (37.9)		
	46-55 years	3 (4.4)	5 (14.3)	8 (7.8)		

Parity		2.23 ± 1.54 (mean ± SD)	1.69 ± 1.45 (mean ± SD)			0.14

BMI		32.34 ± 9.29 (mean ± SD)	30.74 ± 5.98			0.77

Education	Primary	4 (5.9)	(mean ± SD 0(0)	4 (3.9)	5.59	0.061
	Secondary	28 (41.2)	9 (25.7)	37 (35.9)		
	Tertiary	35(51.5)	26(74.3)	61 (59.2)		
	Not given	1 (1.5)	0 (0)	1 (1)		

Marital status	Single	2 (2.9)	2 (5.7)	4 (3.9)	0.49	0.48
	Married	65 (95.6)	32 (91.4)	97 (94.2)		
	Not given	1 (1.5)	1 (2.9)	2 (1.9)		
					1.05	0.59

Religion	Christian	66 (97.1)	35 (100)	101 (98.0)		
	Judaism	1 (1.5)	0 (0)	1 (1)		
	Sabbath	1 (1.5)	0 (0)	1 (1)		

Occupation	Business	29 (42.6)	9 (25.7)	38 (36.9)	4.99	0.28
	Student	8 (11.8)	5 (14.3)	13(12.6)		
	Civil servant	19 (27.9)	15 (42.9)	34 (33)		
	House wife	2 (2.9)	3 (8.6)	5 (4.9)		
	Applicant	3 (4.4)	1 (2.9)	4 (3.9)		
	Not given	7 (10.3)	2 (5.7)	9 (8.7)		

**Table 2:** Comparison of gestational age at insertion between the indication groups.

Indication	GA at insertion (Mean ± STD)	Mean rank	*U* value	*p* value	Shapiro-Wilk stat (sig)
History	14.43 ± 4.32	50.82	1110.00	0.57	0.74 (<0.01)
Ultrasound	14.71 ± 3.59	54.29			

**Table 3:** Comparison of cervical length before cerclage among history and ultrasound indicated patients.

Indication	Cervical length before cerclage (cm) Mean ± STD	Shapiro-Wilk statistics (*p* value)	Mann–Whitney *U* *U* value (*p* value)
History	2.16 ± 0.29	0.62 (<0.01)	98.50 (0.48)
Ultrasound	2.20 ± 0.60		

**Table 4:** Association between indications and outcomes of pregnancy.

Pregnancy outcome	Total *n* (%)	History *n* (%)	Ultrasound *n* (%)	RR	95% CI	*p* value
Miscarriage	12 (11.7)	8 (11.8)	4 (11.40)	1.8571	0.5255 to 6.5629	0.3365
Preterm birth	24 (23.3)	18 (26.50)	6 (17.10)	1.9286	0.8463 to 4.3948	0.1181
Term birth	67 (65.1)	42 (61.80)	25 (71.40)	0.8182	0.4189 to 1.5982	0.587
Total	103 (100.0)	68 (66.0)	35 (34.0)			

**Table 5:** Comparison of birthweight between the research groups.

Groups	Birthweight Mean ± STD	Shapiro-Wilk stat (*p* value	*p* value
History indicated	2.53 ± 0.74	0.93 (<0.01)	0.48
Ultrasound indicated	2.67 ± 0.99		

## Data Availability

On genuine request, the data for this manuscript will be made available.
